# Family Caregiver Strategies to Manage Medication Refusals: Results from a Cross-Sectional Survey

**DOI:** 10.1093/geroni/igaf122.4117

**Published:** 2025-12-31

**Authors:** Reed Bratches, Macy Stockdill, Alejandra Martinez, Elisha Underwood, Paul Barr, Frank Puga, J Nicholas Odom, Rita Jablonski

**Affiliations:** University of Alabama at Birmingham, Birmingham, Alabama, United States; University of Alabama at Birmingham, Birmingham, Alabama, United States; Dartmouth College, Hanover, New Hampshire, United States; University of Alabama at Birmingham, Birmingham, Alabama, United States; Dartmouth College, Hanover, New Hampshire, United States; University of Alabama at Birmingham, Birmingham, Alabama, United States; University of Alabama at Birmingham, Birmingham, Alabama, United States; University of Alabama at Birmingham, Birmingham, Alabama, United States

## Abstract

Family caregivers of persons living with dementia commonly navigate care-resistant behaviors when administering medications. Nonpharmacologic strategies are recommended to manage care-resistant behaviors, but the specific strategies used by family caregivers and their association with caregiver factors including burden, mental health, and perceived medication hassles is unknown. We conducted a survey of 63 family caregivers of persons living with dementia to determine the nonpharmacologic strategies used to manage medication-related care-resistant behaviors and their associations with caregiver factors like burden and mental health. We analyzed strategies descriptively and using univariate statistical tests (t-test and chi-square as appropriate) and used logistic regression to determine strength and direction of statistically significant associations. We found that common strategies included waiting and trying again later (79.4%), covertly administering medications (46.0%), and doing nothing/skipping the medication dose (25.4%). In multivariable regression modeling controlling for demographic factors, we found that higher caregiver mental health scores according to the PROMIS-10 was associated with a decreased likelihood (OR 0.64; 95%CI 0.44, 0.88; p = 0.01) of covertly administering medications. The lower likelihood of covert administration in higher mental health scores indicates the potential for caregiver mental health interventions to affect responses to care-resistant behaviors. Given the cross-sectional nature of our study, additional work is needed to determine the causal pathways between the identified care-resistant behaviors, the strategies used to manage them, and caregiver outcomes, and to test interventions to support caregiver management of medication-related care-resistant behaviors.

